# Imputed expression of schizophrenia‐associated genes and cognitive measures in patients with schizophrenia

**DOI:** 10.1002/mgg3.1942

**Published:** 2022-04-30

**Authors:** Chiara Fabbri, Gian Marco Leggio, Filippo Drago, Alessandro Serretti

**Affiliations:** ^1^ Department of Biomedical and Neuromotor Sciences University of Bologna Bologna Italy; ^2^ Institute of Psychiatry, Psychology & Neuroscience King's College London London UK; ^3^ Department of Biomedical and Biotechnological Sciences, Section of Pharmacology University of Catania Catania Italy

**Keywords:** cognition, gene expression, memory, processing speed, schizophrenia

## Abstract

**Background:**

Cognitive dysfunction is a core manifestation of schizophrenia and one of the best predictors of long‐term disability. Genes increasing risk for schizophrenia may partly act through the modulation of cognition.

**Methods:**

We imputed the expression of 130 genes recently prioritized for association with schizophrenia, using PsychENCODE variant weights and genotypes of patients with schizophrenia in CATIE. Processing speed, reasoning, verbal memory, working memory, vigilance, and a composite cognitive score were used as phenotypes. We performed linear regression models for each cognitive measure and gene expression score, adjusting for age, years of education, antipsychotic treatment, years since the first antipsychotic treatment and population principal components.

**Results:**

We included 425 patients and expression scores of 91 genes (others had no heritable expression; Bonferroni corrected alpha = 5.49e‐4). No gene expression score was associated with cognitive measures, though *ENOX1* expression was very close to the threshold for verbal memory (*p* = 6e‐4) and processing speed (*p* = 7e‐4). Other genes were nominally associated with multiple phenotypes (*MAN2A1* and *PCGF3*).

**Conclusion:**

A better understanding of the mechanisms mediating cognitive dysfunction in schizophrenia may help in the definition of disease prognosis and in the identification of new treatments, as the treatment of cognitive impairment remains an unmet therapeutic need.

## Graphical abstract

The expression of key genes may be associated with cognitive functioning in schizophrenia. This study found that *ENOX1* expression may be associated with verbal memory and processing speed in patients with schizophrenia. *MAN2A1* and *PCGF3* expression levels represent other promising biomarkers.

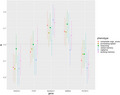

## 1 INTRODUCTION

Schizophrenia is the most common psychotic disorder and it is a leading cause of disability worldwide in people between 25 and 49 years (Vos et al., 2020). Cognitive dysfunction is one of the core manifestations of schizophrenia, as almost all patients with the disease have such impairment, and it is one of the first clinical manifestations or it is present in the prodromal phase (Tripathi et al., 2018). Cognitive functioning is highly associated with social and work functioning, and it is considered one of the best predictors of long‐term disability (Fett et al., 2011; Gold et al., 2002; Harvey & Strassnig, 2012); therefore, it represents an important factor for the definition of prognosis.

Cognitive deficits in schizophrenia involve working memory, attention, processing speed, visual and verbal learning, reasoning, planning, abstract thinking, and problem solving; the corresponding disrupted circuits are the cortico‐cerebellar‐thalamic‐cortical circuits (Tripathi et al., 2018). Evidence of efficacy of antipsychotics in the treatment of cognitive symptoms is limited, and antipsychotic augmentation with cognitive enhancers does not seem to have clinically relevant benefits (e.g., with α7nAChR agonists; Recio‐Barbero et al., 2021). Therefore, the treatment of cognitive deficits remains an unmet need in the therapy of schizophrenia.

Genome‐wide association studies (GWAS) found that schizophrenia and cognitive performance/intelligence share genetic susceptibility loci (Smeland & Andreassen, 2018). Interestingly, the polygenic risk score (PRS) of intelligence (IQ) and educational attainment are positively associated with cognition in schizophrenia, but the PRS of IQ may explain less variance in cognition in schizophrenia than in the general population, leading to the hypothesis that the modulation of cognition in schizophrenia may also depend on specific factors (Richards et al., 2019). There is still poor knowledge about the specific genetic factors involved, as the mentioned study did not identify any genome‐wide significant result, though it analyzed one of the largest schizophrenia samples with available cognitive measures (*n* = 3034). Another GWAS including cases with schizophrenia or bipolar disorder did not report significant loci (Harvey et al., 2020). Findings of a GWAS on a smaller sample including 726 patients with schizophrenia and 667 healthy controls suggested that variants in a few genes may modulate cognitive phenotypes: *NRG3* (neuregulin 3) for abstraction and mental flexibility, *SHANK3* (SH3 And Multiple Ankyrin Repeat Domains 3) and *HCN1* (hyperpolarization activated cyclic nucleotide gated potassium channel 1) for spatial memory (Greenwood et al., 2019). Other studies focused on the role of single genes in cognitive function in patients with schizophrenia (e.g., dysbindin‐1 and dopamine D3 receptor genes; Leggio et al., 2021; Waddington et al., 2020).

Studies that investigated if gene expression may modulate cognition in schizophrenia are even scarcer. Using a gene expression array in a relatively small sample (*n* = 190), the expression of 76 genes was associated with verbal learning and memory, 43 of which also showed differential expression in schizophrenia patients compared with controls (Zheutlin et al., 2016). Other studies focused on the expression of candidate genes (e.g., Ohi et al., 2015; Ruiz‐Sánchez et al., 2021).

The knowledge about the circuits involved in cognitive functioning has guided the development of cognitive enhancers, which act for example through the modulation of glutamatergic, GABAergic, or cholinergic neurotransmission, but they have no evidence of clinical benefits, as previously noted (Recio‐Barbero et al., 2021). A better understanding of the biological mechanisms involved in cognitive dysfunction in schizophrenia might help in finding new treatment targets. The study of changes in gene expression may represent a promising approach, as it can suggest plausible pathogenetic mechanism(s).

The aim of this study was to test if the imputed expression of prioritized genes spanning loci associated with schizophrenia are associated with cognitive phenotypes in patients with schizophrenia included in the Clinical Antipsychotic Trials of Intervention Effectiveness (CATIE) study.

## 2 MATERIALS AND METHODS

### 2.1 Ethical compliance

The study was approved by the institutional review board at each site, and written informed consent was obtained from the patients or their legal guardians. ClinicalTrials.gov identifier (NCT number): NCT00014001.

### 2.2 Sample

The Clinical Antipsychotic Trials of Intervention Effectiveness (CATIE) study was a multi‐centric, double‐blind study to evaluate the relative effectiveness of perphenazine compared to several second‐generation antipsychotics (olanzapine, quetiapine, risperidone, and ziprasidone), randomly assigned in phase 1. In phase 2, patients who stopped the first treatment due to lack of efficacy were randomly assigned to either clozapine or an atypical antipsychotic different from the one prescribed in phase 1. Those who stopped the first antipsychotic due to side effects were randomly assigned to receive either ziprasidone or an atypical medication different from the one received in phase 1. In phase 3, clinicians helped patients to select an open‐label treatment based on experiences in phases 1 and 2. Included individuals provided written informed consent and the study received ethical approval; detailed information about CATIE was previously reported (Stroup et al., 2003).

### 2.3 Phenotypes

Neurocognitive tests were performed in CATIE, as previously described (Keefe et al., 2003). Eleven neurocognitive tests were performed, resulting in 24 individual scores reduced to nine neurocognitive outcome measures, five domain scores and a composite neurocognitive score, as detailed in a previous publication (Keefe et al., 2006). The five domains consisted of processing speed, reasoning, verbal memory, vigilance, and working memory. A factor analysis showed that a single‐factor model comprised of five domain scores was the best fit. The correlations among the factors were medium to high, and scores on individual factors were very highly correlated with the composite neurocognitive score (Keefe et al., 2006). In the present study, we used as phenotypes the five mentioned domains and the composite neurocognitive score; scores were standardized (mean = 0 and standard deviation = 1), to make results comparable among different cognitive measures and easily interpretable. Higher scores in each domain indicated better cognitive functioning. Further information on the neurocognitive assessment battery used in CATIE and on the calculation of the scores in each cognitive domain is in the Supporting Methods.

As we were not interested in evaluating the effect of treatment on cognition, but in the estimation of the level of cognitive functioning in each individual, we included participants who had at least two neurocognitive assessments during follow‐up, and considered the average among the neurocognitive scores in each domain and in the cumulative score, similarly to a previous study (Fabbri & Serretti, 2017).

### 2.4 Gene expression scores

We considered 130 genes spanning loci associated with schizophrenia (Table S1), which had been prioritized by the last Psychiatric Genomics Consortium (PGC) schizophrenia GWAS, based on variant function, location, eQTL (expression quantitative trait locus) effects, and summary‐based Mendelian randomization (The Schizophrenia Working Group of the Psychiatric Genomics Consortium et al., 2020).

In CATIE, 738 participants were genotyped using the Affymetrix 500 K and Perlegen's custom 164 K chip (Sullivan et al., 2008). Quality control of genotypes was performed as described in a previous study (Fabbri & Serretti, 2017). Imputation of genotypes was performed using the Haplotype Reference Consortium r1.1 2016 panel, as reported in the same study. Further details are reported as Supporting Methods.

We imputed the expression of the genes of interest using individual‐level genotypes and PsychENCODE variant weights. Variant weights were based on the correlation between genetic variants and gene expression in the PsychENCODE brain samples (dorsolateral prefrontal cortex, DLPFC, obtained in ~1000 subjects), considering linkage disequilibrium between variants (PsychENCODE Consortium et al., 2015). Weights were in FUSION format, which includes several models to estimate different genetic architectures (best linear unbiased prediction [BLUP], elastic net, lasso, Bayesian Sparse Linear Mixed Model [BSLMM], and top SNPs; Gusev et al., 2016). We selected the weights corresponding to the model with the highest cross‐validation coefficient of determination (CV R2). We assumed an additive model, where gene expression was estimated for each gene as the sum of the number of alleles carried by each participant multiplied by the corresponding weight at each locus; scores were calculated using PLINK 1.9 (Chang et al., 2015).

### 2.5 Statistical analysis

We fitted linear regression models to investigate the possible association between cognitive phenotypes and gene expression scores, using R version 4.1.0. All the analyses were adjusted for age, years of education, number of antipsychotics at baseline, years since the first antipsychotic treatment, and ancestry‐relevant population principal components (first four population principal components, as previously described; Fabbri & Serretti, 2017). The Bonferroni correction was applied to correct for multiple testing, considering the total number of genes tested, while not the six phenotypes considered, as they were moderately highly correlated among each other (Keefe et al., 2006). Keeping in mind this point, we decided to test if significant associations with specific cognitive domains may have an effect independent from the composite cognitive score, by adding this variable to the model as covariate.

## 3 RESULTS

Gene expression was imputed in 479 patients of European ancestry (369 males, 110 females, mean age 41.13 ± 11.49), other sample characteristics were previously reported (Fabbri & Serretti, 2017; see also Table S2 and Figure S1). Cognitive phenotypes were available in 425 out of 479 individuals. We considered expression scores of 91 among the 130 genes prioritized by the PGC (The Schizophrenia Working Group of the Psychiatric Genomics Consortium et al., 2020), as no significant heritability of gene expression was found in PsychENCODE for some of the genes (Table S1). Based on the number of analyzed genes, a Bonferroni corrected *p* value of 5.49e‐4 was considered as the significance threshold.

**FIGURE 1:**
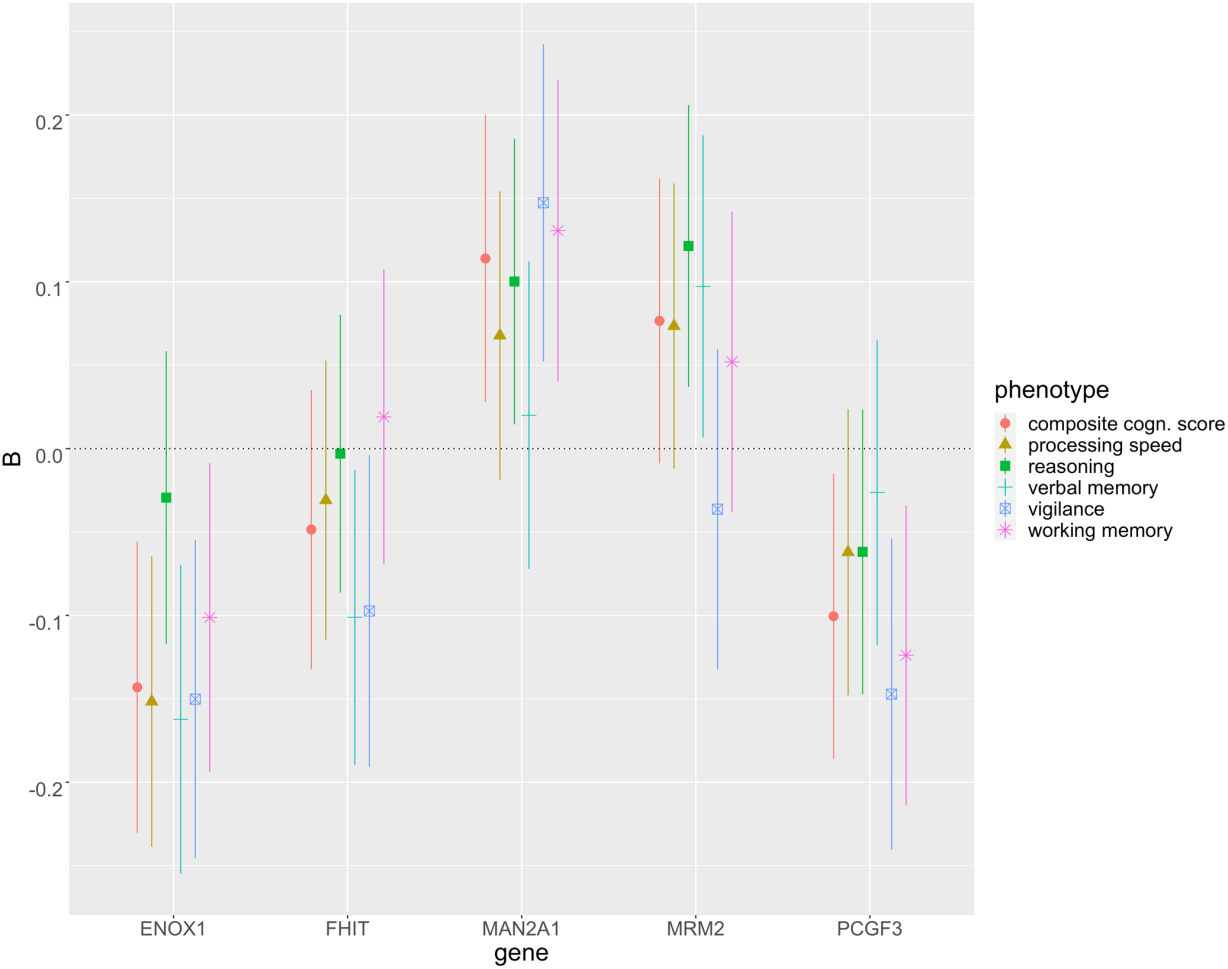
Regression coefficient (B) and 95% confidence intervals for the top genes in terms of association with cognitive measures (see also Table 1)

*ENOX1* (OMIM ID: 610914) expression was very close to the significance threshold for being negatively associated (i.e., higher gene expression = worse cognitive functioning) with processing speed (*p* = 7e‐4) and verbal memory (*p* = 6e‐4). This gene showed nominal associations (*p* < 0.05) also with vigilance (*p* = 0.002), working memory (*p* = 0.03), and the global cognitive score (*p* = 0.001), in the same direction (Table 1, Figure 1).

**TABLE 1:** Genes which imputed expression scores were associated at least at the nominal level (*p* < 0.05) with two or more cognitive phenotypes

Gene symbol (OMIM ID)	Phenotype	B (SE)	95% CI	*p*	Neuropsychiatric phenotypes associated with variants in the gene in previous GWASs^a^
*ENOX1* (610914)	Processing speed	−0.15 (0.04)	−0.24/−0.06	7e‐4	Unipolar depression, bipolar disorder, cross‐disorder effect across psychiatric disorders
	Verbal memory	−0.16 (0.05)	−0.25/−0.07	6e‐4	
	Vigilance	−0.15 (0.05)	−0.25/−0.05	0.002	
	Working memory	−0.10 (0.05)	−0.19/−0.01	0.03	
	Composite cognitive score	−0.14 (0.04)	−0.23/−0.06	0.001	
*MAN2A1* (154582)	Reasoning	0.10 (0.04)	0.01/0.19	0.02	Intelligence, years of education, educational attainment, highest math class taken, cognitive performance, smoking initiation
	Working memory	0.13 (0.05)	0.04/0.22	0.005	
	Vigilance	0.15 (0.05)	0.05/0.24	0.003	
	Composite cognitive score	0.11 (0.04)	0.03/0.20	0.01	
*PCGF3* (617543)	Working memory	−0.12 (0.05)	−0.21/−0.03	0.007	Intelligence, self‐reported educational attainment, mathematical ability, cognitive function
	Vigilance	−0.15 (0.05)	−0.24/−0.05	0.002	
	Composite cognitive score	−0.10 (0.04)	−0.19/−0.02	0.02	
*MRM2* (606906)	Reasoning	0.12 (0.04)	0.04/0.21	0.005	–
	Verbal memory	0.10 (0.05)	0.01/0.19	0.04	
*FHIT* (601153)	Verbal memory	−0.10 (0.05)	−0.19/−0.01	0.03	Unipolar depression, depressive symptoms, sleep duration, wellbeing, neuroticism, autism spectrum disorders, self‐reported educational attainment, smoking behavior, response to placebo
	Vigilance	−0.10 (0.05)	−0.19/−0.004	0.04	

Abbreviation: CI, confidence interval.

^a^ Phenotypes other than schizophrenia, associated at the genome‐wide significance level (*p* < 5e‐8), according to GWAS catalog: https://www.ebi.ac.uk/, accessed on 15 June 2021; GWAS catalog annotations are based on the last Ensembl release, genes in which a variant maps are reported or the closest upstream or downstream gene within 50 kb.

Though these results were not significant after Bonferroni correction, we mention that imputed expression scores of two genes were nominally associated with three or four cognitive phenotypes. In detail, *MAN2A1* (OMIM ID: 154582) expression was positively associated with reasoning (*p* = 0.02), working memory (*p* = 0.005), vigilance (*p* = 0.003), and the global cognitive score (*p* = 0.01; i.e., higher gene expression = higher cognitive functioning). *PCGF3* (OMIM ID: 617543) expression was negatively related with working memory (*p* = 0.007), vigilance (*p* = 0.002) and the global cognitive score (*p* = 0.02). Genes showing nominal associations with two cognitive phenotypes were *MRM2* (alias: *FTSJ2*, OMIM ID: 606906) and *FHIT* (OMIM ID: 601153), with positive and negative nominal associations, respectively, see Table 1 and Figure 1.

After adjusting the models for the composite cognitive score, none of the associations previously reported involving *ENOX1* had *p* < 0.05, while *MAN2A1* and *PCGF3* gene expression had a nominal association with vigilance only, *MRM2* with reasoning, and the previously observed associations between *FHIT* and cognitive phenotypes had still nominal significance. The associations that maintained nominal significance were in the same direction reported in the analyses without including the composite cognitive score as covariate.

The results of the analyses for all genes are described in Table S3, while the results of the multi‐variate analyses including the composite cognitive score as covariate are in Table S4.

## 4 DISCUSSION

Genes increasing risk for schizophrenia may partly act through the modulation of processes relevant to cognitive functions, as cognitive impairment is a core manifestation of the disease (Tripathi et al., 2018). With the aim of contributing to test this hypothesis, we studied if imputed gene expression of genes spanning loci associated with schizophrenia in the largest case–control GWAS are correlated with cognitive phenotypes in patients with schizophrenia.

Our top finding was *ENOX1* (ecto‐NOX disulfide‐thiol exchanger 1), which expression was negatively associated with cognitive performance in several domains at the nominal level (i.e., higher expression = worse cognitive functioning), and it was very close to the Bonferroni significance threshold for processing speed and verbal memory (Table 1, Figure 1). The protein encoded by this gene is involved in plasma membrane electron transport pathways and modulates many important cellular functions, such as cell growth and survival, intracellular redox homeostasis, cytoskeletal reorganization, and neurite outgrowth (Scarlett et al., 2005; Wang et al., 2013). *ENOX1* inhibitors were studied for their anti‐angiogenic properties, potentially useful for treating cancer (Venkateswaran et al., 2013), but the possible effect of this type of drug on cognitive function has not been explored.

In addition to associations with psychiatric disorders (Table 1), a couple of previous studies reported associations between *ENOX1* variants and cognition. A variant in *ENOX1* (rs7491050:T > C) was found as one of the top variants modulating amplitude of low‐frequency fluctuations in the medial prefrontal cortex and gray matter volume in thalamus, putamen and bilateral temporal gyrus, that were associated with working memory (Luo et al., 2018). Nominal associations were reported between *ENOX1* (variant rs4143229:C > A,G,T), processing speed and language (vocabulary) in Generation Scotland (Meijsen et al., 2018).

*MAN2A1* (mannosidase alpha class 2A member 1) expression was positively associated with multiple cognitive phenotypes (i.e., higher expression was associated with better cognitive functioning), but none was significant after Bonferroni correction. This gene encodes for a glycosyl hydrolase, a critical enzyme in development, as its deletion in animal models causes infertility, autoimmune disorders, and neurologic abnormalities (Mealer et al., 2020). As reported in Table 1, variants in this gene have been associated with several cognition‐related measures in GWASs, such as intelligence, years of education, educational attainment, and highest math class taken.

*PCGF3* (polycomb group ring finger 3) expression showed associations of similar significance to *MAN2A1*, but in the opposite direction (i.e., higher expression had a negative effect on cognitive phenotypes). The corresponding protein is involved in the regulation of gene expression and neuronal differentiation (Monderer‐Rothkoff et al., 2021). *PCGF3* has also been associated with multiple cognitive phenotypes (Table 1).

Other two genes reported in Table 1 (*MRM2* and *FHIT*) had more marginal nominal associations with cognition and poor support of being involved in the modulation of cognitive functions in the previous literature.

We tested if any of the reported nominal associations survived after adjusting the model also for the compositive cognitive score, to evaluate if some effects may be independent from the general cognitive functioning. We found that mostly associations with vigilance showed *p* < 0.05 in these analyses, suggesting that this phenotype is less correlated with the composite cognitive score compared to other cognitive domains; it was indeed previously reported that in CATIE the composite score had higher correlation with processing speed and working memory than other cognitive scores, including vigilance (Keefe et al., 2006).

The limitations of the present study should be considered. First, we calculated imputed and not measured gene expression, with the advantage of a much easier, faster, and more scalable approach compared to RNA arrays or sequencing, but without an actual measurement of gene expression. PsychENCODE weights are referred to the DLPFC only and they estimate cis‐eQTL effects, while trans‐eQTL effects are not considered. We decided to not use weights obtained using other samples such as GTEx (GTEx Consortium, 2015), that includes also other brain tissues, to reduce the issue of multiple testing by prioritizing the largest sample (i.e., PsychENCODE). Our analyses were focused on individuals of European ancestry, who represented the largest proportion of CATIE's participants and corresponded to the ancestry of individuals in PsychENCODE.

## 5 CONCLUSIONS

Our study identified a few genes which expression may be associated with cognitive functioning in patients with schizophrenia, thought the associations did not survive after multiple‐testing correction. Our approach can be easily applied to other samples with schizophrenia‐spectrum disorders, to validate and expand the present findings, clarifying the mechanisms mediating cognition in schizophrenia, and identify new possible targets for pharmacological interventions, as cognitive dysfunction remains one of the main unmet therapeutic needs in schizophrenia.

## CONFLICT OF INTEREST

Alessandro Serretti is or has been consultant/speaker for: Abbott, Abbvie, Angelini, Astra Zeneca, Clinical Data, Boheringer, Bristol Myers Squibb, Eli Lilly, GlaxoSmithKline, Innovapharma, Italfarmaco, Janssen, Lundbeck, Naurex, Pfizer, Polifarma, Sanofi, Servier, and Taliaz. Chiara Fabbri was a speaker for Janssen. The other authors declare no potential conflicts of interest.

## AUTHOR CONTRIBUTIONS

Chiara Fabbri designed the study with support from the other co‐authors, Chiara Fabbri performed the statistical analysis and wrote the first draft of the paper; Alessandro Serretti supervised the work; Alessandro Serretti, Gian Marco Leggio, and Filippo Drago contributed to revisions that led to the final version of the work.

## ETHICS APPROVAL

The CATIE study was approved by the institutional review board at each site, and written informed consent was obtained from the patients or their legal guardians. ClinicalTrials.gov identifier (NCT number): NCT00014001.

## Supporting information


Appendix S1

Figure S1

Tables S1‐S4

## Data Availability

Data collected within the CATIE study are available to researchers with an approved project through the NIMH Human Genetics Initiative http://nimhgenetics.org/.
