# Supplementary material for: Imputed expression of schizophrenia‐associated genes and cognitive measures in patients with schizophrenia
Source: Mol Genet Genomic Med. 2022 Apr 30;10(6):e1942. doi: 10.1002/mgg3.1942 (PMC9184669; doi:10.1002/mgg3.1942)
Supplement: Supplementary file 1 — Appendix S1 [file MGG3-10-e1942-s001.docx]

**Imputed expression of schizophrenia-associated genes and cognitive measures**

**in patients with schizophrenia**

**Supplementary Methods**

**Neurocognitive assessment battery in CATIE**

A battery consisting of 11 tests was administered at each neurocognitive visit (baseline, 2 months, 6 months, and 18 months or end of study), requiring approximately 90 minutes (Keefe et al., 2003). The following cognitive domains were evaluated:

- Verbal fluency: was assessed using the Controlled Oral Word Association Test and Category Instances test (Benton & Hamscher, 1978). In the first, individuals were asked to generate as many words as possible that start with a given letter in three trials of 60 seconds each, while in the second subjects had 60 seconds to generate as many words as possible within each of three categories. For both tests, the number of correct words in each category is recorded.
- Working memory: was evaluated by the Letter-Number Span test of auditory working memory (Gold et al., 1997) and the Computerized test of visuospatial working memory (Hershey et al., 1999). The first test consists in reordering clusters of letters combined with numbers that are presented orally, the second measures subjects' ability to remember the position of a cue on a screen. The number of correct sequences and the mean error in millimeters (distance between recall and actual target) are measured, respectively.
- Verbal learning and memory: was measured using the Hopkins Verbal Learning Test (Brandt, 1991), which consists of 12 nouns read aloud for three trials, each trial followed by a free-recall test. The number of words correctly recalled in each trial is recorded.
- Social cognition: was assessed with the Face Emotion Discrimination Task (FEDT) (Kerr & Neale, 1993), a test that evaluates the ability to determine if two faces presented next to one another are expressing the same emotion; the number of faces correctly classified is counted.
- Motor function: was evaluated using the Grooved Pegboard test (Lafayette Instrument Company, 1989) and WAIS-R Digit Symbol Test (Wechsler, 1974). In the first, participants have to insert in a specified order 25 pegs into a pegboard; in the second, each number from 1 to 9 is associated with a different shape, individuals are given a list of numbers and are asked to copy as many shapes associated with them as possible in 90 seconds. The measures are the number of pegs successfully inserted and number of shapes copied, respectively.
- Attention: was assessed by the Computerized Continuous Performance Test (CPT) (Cornblatt et al., 1988), which measures the ability of the subject to respond to the repetition of a two-digit, three or four digit number.
- Executive function: was measured with the Computerized Wisconsin Card Sorting Test (WCST) (Heaton et al., 1993) and WISC-III mazes (Wechsler, 1991). In the WCST, participants are presented with cards that they have to match, but without knowing the rule to match; however, they are told if a particular match is correct or not. The WISC-III evaluates the performance to draw through a series of nine mazes without entering blind alleys.

**Neurocognitive scores**

In the present study, we considered five cognitive domains and a composite neurocognitive score, in accordance with (Keefe et al., 2006), and available as part of the CATIE dataset. Scores in each cognitive domain were calculated as follows:

- Processing speed: standardized sum of results of tests assessing verbal fluency and motor function.
- Reasoning: standardized sum of results of tests measuring executive function.
- Verbal memory: standardized results of the Hopkins Verbal Learning Test.
- Vigilance: standardized results of the CPT test.
- Working memory: standardized sum of results of working memory tests.

The neurocognitive composite score was the standardized sum of the five described cognitive domains.

**Genotyping, quality control and imputation in CATIE**

In CATIE, 738 participants were genotyped by Perlegen Sciences using the Affymetrix 500K and Perlegen's custom 164K chip (Sullivan et al., 2008).

Genotypes were imputed with the Haplotype Reference Consortium (HRC version r1.1 2016) panel as reference, using the Michigan imputation Server (https://imputationserver.sph.umich.edu/start.html).

Pre-imputation quality control was carried out according to the following criteria: 1) variants with missing rate ≥ 5%; 2) monomorphic variants; 3) subjects with genotyping rate < 97%; 4) subjects with gender discrepancies; 5) subjects with abnormal heterozygosity; 6) related subjects (identity by descent (IBD) >0.1875 (Anderson et al., 2010); 7) population outliers according to Eigensoft analysis of linkage-disequilibrium-pruned genetic data (Patterson et al., 2006); 8) non-white subjects. Hardy–Weinberg equilibrium was not used as an exclusion criterion, since departures from Hardy–Weinberg equilibrium are expected in a case-only study (Wittke-Thompson et al., 2005)

Post-imputation quality control was performed according to the following criteria: 1) poor imputation quality (R2 < 0.30 (Li et al., 2010) (Pistis et al., 2015)); 2) minor allele frequency (MAF) < 0.01.

**References**

Anderson, C. A., Pettersson, F. H., Clarke, G. M., Cardon, L. R., Morris, A. P., & Zondervan, K. T. (2010). Data quality control in genetic case-control association studies. *Nature Protocols*, *5*(9), 1564–1573. https://doi.org/10.1038/nprot.2010.116

Benton, A., & Hamscher, K. (1978). *Multilingual Aphasia Examination Manual (revised)* (University of Iowa).

Brandt, J. (1991). The hopkins verbal learning test: Development of a new memory test with six equivalent forms. *Clinical Neuropsychologist*, *5*(2), 125–142. https://doi.org/10.1080/13854049108403297

Cornblatt, B. A., Risch, N. J., Faris, G., Friedman, D., & Erlenmeyer-Kimling, L. (1988). The Continuous Performance Test, identical pairs version (CPT-IP): I. New findings about sustained attention in normal families. *Psychiatry Research*, *26*(2), 223–238. https://doi.org/10.1016/0165-1781(88)90076-5

Gold, J. M., Carpenter, C., Randolph, C., Goldberg, T. E., & Weinberger, D. R. (1997). Auditory working memory and Wisconsin Card Sorting Test performance in schizophrenia. *Archives of General Psychiatry*, *54*(2), 159–165. https://doi.org/10.1001/archpsyc.1997.01830140071013

Heaton, R., Chelune, G., Taley, J., Kay, G., & Curtiss, G. (1993). *Wisconsin Card Sorting Test Manual: Revised and Expanded*. Psychological Assessment Resources.

Hershey, T., Selke, G., Fucetola, R., & Newcomer, J. (1999). *Spatial long-term memory but not working memory decreases over time in schizophrenia*. *25(Part 1)*, 572.

Keefe, R. S. E., Bilder, R. M., Harvey, P. D., Davis, S. M., Palmer, B. W., Gold, J. M., Meltzer, H. Y., Green, M. F., Miller, D. D., Canive, J. M., Adler, L. W., Manschreck, T. C., Swartz, M., Rosenheck, R., Perkins, D. O., Walker, T. M., Stroup, T. S., McEvoy, J. P., & Lieberman, J. A. (2006). Baseline Neurocognitive Deficits in the CATIE Schizophrenia Trial. *Neuropsychopharmacology*, *31*(9), 2033–2046. https://doi.org/10.1038/sj.npp.1301072

Keefe, R. S. E., Mohs, R. C., Bilder, R. M., Harvey, P. D., Green, M. F., Meltzer, H. Y., Gold, J. M., & Sano, M. (2003). Neurocognitive assessment in the Clinical Antipsychotic Trials of Intervention Effectiveness (CATIE) project schizophrenia trial: Development, methodology, and rationale. *Schizophrenia Bulletin*, *29*(1), 45–55. https://doi.org/10.1093/oxfordjournals.schbul.a006990

Kerr, S. L., & Neale, J. M. (1993). Emotion perception in schizophrenia: Specific deficit or further evidence of generalized poor performance? *Journal of Abnormal Psychology*, *102*(2), 312–318. https://doi.org/10.1037//0021-843x.102.2.312

Lafayette Instrument Company. (1989). *Grooved Pegboard Instruction Manual, Model 32025.* (Lafayette, IN). Lafayette Instrument Company.

Li, Y., Willer, C. J., Ding, J., Scheet, P., & Abecasis, G. R. (2010). MaCH: Using sequence and genotype data to estimate haplotypes and unobserved genotypes. *Genetic Epidemiology*, *34*(8), 816–834. https://doi.org/10.1002/gepi.20533

Patterson, N., Price, A. L., & Reich, D. (2006). Population structure and eigenanalysis. *PLoS Genetics*, *2*(12), e190. https://doi.org/10.1371/journal.pgen.0020190

Pistis, G., Porcu, E., Vrieze, S. I., Sidore, C., Steri, M., Danjou, F., Busonero, F., Mulas, A., Zoledziewska, M., Maschio, A., Brennan, C., Lai, S., Miller, M. B., Marcelli, M., Urru, M. F., Pitzalis, M., Lyons, R. H., Kang, H. M., Jones, C. M., … Sanna, S. (2015). Rare variant genotype imputation with thousands of study-specific whole-genome sequences: Implications for cost-effective study designs. *European Journal of Human Genetics: EJHG*, *23*(7), 975–983. https://doi.org/10.1038/ejhg.2014.216

Sullivan, P. F., Lin, D., Tzeng, J.-Y., van den Oord, E., Perkins, D., Stroup, T. S., Wagner, M., Lee, S., Wright, F. A., Zou, F., Liu, W., Downing, A. M., Lieberman, J., & Close, S. L. (2008). Genomewide association for schizophrenia in the CATIE study: Results of stage 1. *Molecular Psychiatry*, *13*(6), 570–584. https://doi.org/10.1038/mp.2008.25

Wechsler, D. (1974). *Wechsler Adult Intelligence Scale-Revised*. Psychological Corporation.

Wechsler, D. (1991). *Wechsler Intelligence Scale for Children* (3rd ed). Psychological Corporation.

Wittke-Thompson, J. K., Pluzhnikov, A., & Cox, N. J. (2005). Rational inferences about departures from Hardy-Weinberg equilibrium. *American Journal of Human Genetics*, *76*(6), 967–986. https://doi.org/10.1086/430507
