# Supplementary material for: Imputed expression of schizophrenia‐associated genes and cognitive measures in patients with schizophrenia
Source: Mol Genet Genomic Med. 2022 Apr 30;10(6):e1942. doi: 10.1002/mgg3.1942 (PMC9184669; doi:10.1002/mgg3.1942)

Supplementary Figure 1: distribution of the composite cognitive score and the five cognitive domains tested in this study. These measures were available in 425 individuals from CATIE.

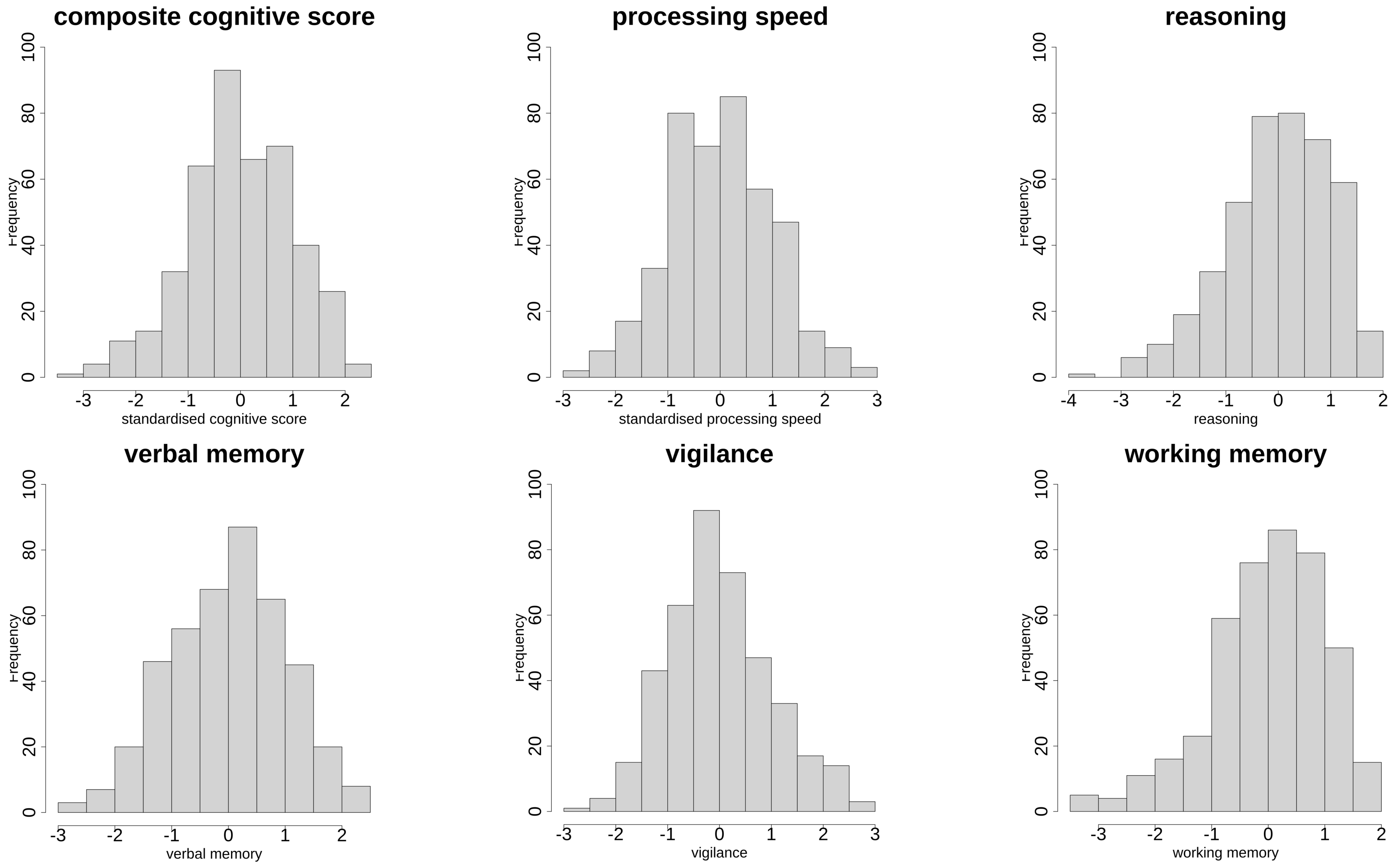

Supplement: Supplementary file 2 — Figure S1 [file MGG3-10-e1942-s002.pdf]
